# Alpha7 Nicotinic Acetylcholine Receptors Play a Predominant Role in the Cholinergic Potentiation of N-Methyl-D-Aspartate Evoked Firing Responses of Hippocampal CA1 Pyramidal Cells

**DOI:** 10.3389/fncel.2017.00271

**Published:** 2017-09-05

**Authors:** Zsolt K. Bali, Lili V. Nagy, István Hernádi

**Affiliations:** ^1^Department of Experimental Zoology and Neurobiology, Faculty of Sciences, University of Pécs Pécs, Hungary; ^2^János Szentágothai Research Center, Center for Neuroscience, University of Pécs Pécs, Hungary

**Keywords:** scopolamine, methyllycaconitine, *in vivo* electrophysiology, muscarinic AChR, alpha7 nicotinic AChR, pharmacological amnesia models, glutamatergic neurotransmission, cholinergic neurotransmission

## Abstract

The aim of the present study was to identify *in vivo* electrophysiological correlates of the interaction between cholinergic and glutamatergic neurotransmission underlying memory. Extracellular spike recordings were performed in the hippocampal CA1 region of anesthetized rats in combination with local microiontophoretic administration of N-methyl-D-aspartate (NMDA) and acetylcholine (ACh). Both NMDA and ACh increased the firing rate of the neurons. Furthermore, the simultaneous delivery of NMDA and ACh resulted in a more pronounced excitatory effect that was superadditive over the sum of the two mono-treatment effects and that was explained by cholinergic potentiation of glutamatergic neurotransmission. Next, animals were systemically treated with scopolamine or methyllycaconitine (MLA) to assess the contribution of muscarinic ACh receptor (mAChR) or α7 nicotinic ACh receptor (nAChR) receptor-mediated mechanisms to the observed effects. Scopolamine totally inhibited ACh-evoked firing, and attenuated the firing rate increase evoked by simultaneous application of NMDA and ACh. However, the superadditive nature of the combined effect was preserved. The α7 nAChR antagonist MLA robustly decreased the firing response to simultaneous application of NMDA and ACh, suspending their superadditive effect, without modifying the tonic firing rate increasing effect of ACh. These results provide the first *in vivo* electrophysiological evidence that, in the hippocampal CA1 region, α7 nAChRs contribute to pyramidal cell activity mainly through potentiation of glutamatergic signaling, while the direct cholinergic modulation of tonic firing is notably mediated by mAChRs. Furthermore, the present findings also reveal cellular physiological correlates of the interplay between cholinergic and glutamatergic agents in behavioral pharmacological models of cognitive decline.

## Introduction

Cholinergic and glutamatergic neurotransmitter receptors are important targets for pharmacological interventions against cognitive impairment. For instance, currently available pharmacological treatments for Alzheimer’s disease are based on either the augmentation of cholinergic neurotransmission via inhibition of acetylcholinesterase enzyme activity (McGleenon et al., 1999), or the modulation of N-methyl-D-aspartate type glutamate receptor (NMDAR) using the weak receptor antagonist memantine. Among the many existing hypotheses (for a review see Parsons et al., 2007), a potential explanation for the procognitive effects of memantine is that memantine increases signal-to-noise ratio of LTP generation by blocking pathological overactivation of NMDARs (Collingridge et al., 2013). Furthermore, agonists and positive allosteric modulators of the α7 nicotinic acetylcholine receptors (nAChRs) are potential novel drug candidates for cognitive enhancement, as they have already been shown in preclinical investigations to produce pronounced improvement of cognitive performance in different behavioral tasks in animals (for a review see Wallace and Porter, 2011). At the same time, the critical role of cholinergic and glutamatergic transmission in normal cognition has been demonstrated by animal models of transient amnesia, which can be induced by antagonists acting on AChRs or NMDARs.

Scopolamine is a muscarinic ACh receptor (mAChR) antagonist that potently impairs performance in a variety of cognitive behavioral tests in rodents and primates (Buccafusco et al., 2008). Though used less frequently, the α7 nAChR antagonist methyllycaconitine (MLA) also reportedly possesses notable amnestic potential, as shown in several memory tests in rodents (Tinsley et al., 2011; Andriambeloson et al., 2014). Apart from blocking cholinergic neurotransmission, an alternative means of pharmacologically inducing amnesia is through antagonism of glutamatergic transmission, for example, by inhibiting NMDAR activity with phencyclidine (Kesner et al., 1983), ketamine (Cannon et al., 2013) or dizocilpine (MK-801; van der Staay et al., 2011). Furthermore, a recent behavioral study from our laboratory conducted on rats has additionally demonstrated the substantial role of cholinergic-glutamatergic receptor interactions in normal cognitive performance in a working memory task (Bali et al., 2015).

While the crucial role of cholinergic and glutamatergic transmission and their interaction in maintaining cognitive performance is obvious from behavioral studies, further investigations are needed to clarify the underlying neuronal and network-level mechanisms in the brain structures known to be involved in cognitive processes. The CA1 region of the hippocampus, for example is associated with declarative and spatial memory (Tsien et al., 1996). Here, α7 nAChRs are located not only on interneurons (Jones and Yakel, 1997), but also on the presynaptic surface of both glutamatergic and gamma-aminobutyric acid (GABA)ergic terminals (Fabian-Fine et al., 2001). *In vitro* studies in hippocampal slices and synaptosomes have shown that activation of α7 nAChRs increases the release of different neurotransmitters including glutamate, glycine and noradrenaline; however, noradrenaline terminals are indirectly stimulated by α7 nAChRs as a consequence of the increased glutamate levels (Barik and Wonnacott, 2006; Zappettini et al., 2010, 2011). The presence of presynaptic α7 nAChRs and their modulatory effects on glutamate release in the hippocampus also highlight the role of cholinergic-glutamatergic interactions in memory, and suggest that the hippocampal CA1 region would be a suitable structure for identifying *in vivo* cellular electrophysiological correlates of such interaction.

For our study, we recorded the extracellular firing activity of rat hippocampal CA1 neurons *in vivo*, and investigated the local effects of separately or simultaneously releasing ACh and NMDA into the vicinity of the neurons. The contribution of α7-nAChR-mediated mechanisms to neuronal responses to locally delivered ACh and NMDA was tested using systemic administration of the selective α7 nAChR antagonist MLA. In this way, we avoided the interfering effects of receptor desensitization that could occur if α7 nAChRs were directly activated with selective agonists (Egan, 1989; Quick and Lester, 2002). Furthermore, for comparison purposes, the mAChR-mediated mechanisms were also investigated by injecting the general mAChR antagonist scopolamine. As both MLA and scopolamine are well-known for their amnestic effects in various cognitive tests of rodents (Andriambeloson et al., 2014), our present research may also provide valuable data on the underlying physiological mechanisms of pharmacologically-induced amnesia models in rodents.

## Materials and Methods

### Animals and Surgical Preparations

This study was approved by the Animal Care Committee of the University of Pécs. Procedures fully complied with Decree No. 40/2013 (II. 14.) of the Hungarian Government and EU Directive 2010/63/EU on the protection of animals used for scientific purposes. Fifteen Wistar rats (six males) weighing between 285 g and 540 g were used in the experiments. Initial anesthesia was achieved with a single chloral hydrate injection (400 mg/kg b.w., i.p.). Stable anesthesia was ensured through continuous intravenous administration of the anesthetic via a jugular vein cannula (initial 100 mg/kg/h dose was adjusted later if needed). Anesthetized rats were placed in a stereotaxic frame, and an incision was made on the scalp. A hole was drilled in the skull and a small part of the dura was removed to access brain tissue. At the end of the stereotaxic surgery, a multi-barrel carbon fiber microelectrode (Carbostar, Kation Scientific Ltd., Minneapolis, MN, USA) was inserted into the CA1 region of the dorsal hippocampus (in conformity with the rat brain atlas by Paxinos and Watson (2014): AP 4.0–4.6, ML 1.9–2.3 from bregma, and DV 2.0–3.4 from dura). The microelectrode consisted of a centrally positioned recording channel made from carbon fiber (~7 μm in diameter) and of 3–6 microiontophoresis glass capillaries (~1 μm in inner tip diameter each) around the recording channel. The borosilicate glass insulation of the recording channel and the microiontophoresis capillaries ended at the same level, and the tip of the carbon fiber extended by ~25 μm from the insulation (Budai and Molnár, 2001; Budai et al., 2010). Tail flick test was performed regularly and local field potentials were monitored throughout the experiments to control the depth of anesthesia.

### Extracellular Recording and Microiontophoresis

The firing activity of CA1 neurons was recorded extracellularly through the central carbon fiber of the microelectrode. The electrophysiological signal was amplified and band-pass filtered between 300 Hz and 2000 Hz by analog electrophysiological amplifiers (BioAmp, Supertech Kft., Pécs, Hungary; NeuroLog, Digitimer Ltd., Welwyn Garden City, UK). The data were digitized at 25 kHz by an analog-to-digital converter (CED Power 1401) using Spike2 software (both manufactured by Cambridge Electronic Design Ltd., Cambridge, UK). Extracellular action potentials (spikes) were defined as stable spike signals exceeding the peak background noise level by at least twice and its root-mean-square by at least five times. Similar to our previous report (Bali et al., 2014), neuronal spikes were sorted in clusters using the template matching algorithm of the Spike2 software. Then, hippocampal pyramidal neurons and interneurons were electrophysiologically separated into complex-spiking and single-spiking neuronal subtypes, respectively (Csicsvari et al., 1998), according to their shape and to the observation of autocorrelograms representing their firing pattern. Complex-spiking neurons typically fired 2–7 spikes with a short interspike-interval. Therefore, spike clusters which showed a sharp peak between ±3 ms–6 ms on the autocorrelogram, were identified as complex-spiking neurons. On the other hand, spike clusters that did not show this property, while showing a gradual increase of firing probability as interspike-interval increases, were identified as single-spiking neurons. Separation processes and inclusion criteria are described in more details in Bali et al. (2014). In the present study, only complex-spiking neurons (pyramidal cells) were analyzed.

Iontophoretic drug delivery was performed using pipettes surrounding the central channel of the microelectrode. A constant current stimulator was used to eject the neuroactive compounds (Neurophore BH-2 System, Medical System Corp., Greenvale, NY, USA). Two of the glass capillaries of the multi-barrel electrode were filled with 50 mM NMDA (Sigma-Aldrich) and 100 mM ACh (Sigma-Aldrich), diluted in distilled water.

**Figure 1:**
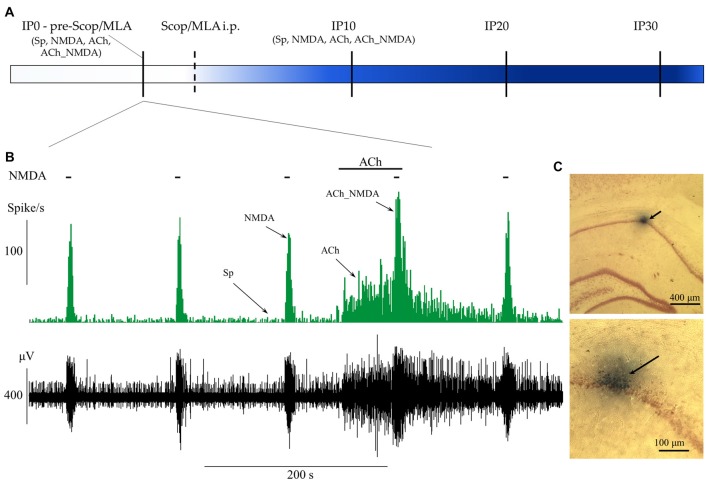
Schematic representation of the experimental paradigm and the protocol of iontophoretic and systemic treatments. **(A)** Timeline of systemic pharmacological treatments and measurement points before (IP0) and 10, 20 and 30 min after systemic administration of scopolamine or methyllycaconitine (MLA; IP10, IP20 and IP30). At each point of measurement, firing frequency values were established for four test conditions (Sp: spontaneous firing rate, NMDA: NMDA-evoked firing rate, ACh: ACh-evoked firing rate, ACh_NMDA: firing rate evoked by simultaneous delivery of NMDA and ACh; for detailed explanation refer to “Extracellular Recording and Microiontophoresis” Section) according to the concurrent local iontophoretic treatments. **(B)** A typical electrophysiological recording that demonstrates the four test conditions. Local deliveries of NMDA and ACh are indicated by horizontal bars above the firing rate histogram (top) and the raw waveform data (bottom). **(C)** Example of histological labeling of recording sites with Chicago Sky Blue 6B dye observed under light microscopy at two different magnifications. The arrows indicate the position of the microelectrode according to the deposition of the microiontophoretically applied dye.

The experimental protocol is represented in Figures 1A,B. During recording sessions, NMDA was periodically ejected by negative constant current (in the range from 10 nA to 75 nA) for 5 s every 2 min, similar to the experimental protocol used in Szegedi et al. (2010) and in our previous study (Bali et al., 2014). After establishing stable responsiveness to NMDA (at least three repeatable excitation peaks), ACh was ejected in regular intervals by positive constant current (in the range from 10 nA to 80 nA) for 70 s. The last phase of ACh ejection overlapped with the next NMDA delivery to test the effects of the combined delivery of the two agents. According to the periodically repeated events of NMDA- and ACh-delivery, four distinct test conditions were designed to examine the physiological and pharmacological properties of the recorded neurons before and after systemic injection of amnestic agents: (1) spontaneous firing in the absence of iontophoretic drug delivery, measured in a 60 s time window immediately before the iontophoresis of NMDA alone (Sp); (2) firing activity during the excitation peak (typically 8 s) evoked by iontophoretic delivery of NMDA (NMDA); (3) firing activity evoked by iontophoretic delivery of ACh, measured in a 60 s time window immediately before the co-administration of NMDA (ACh); and (4) firing activity evoked by the simultaneous iontophoretic delivery of ACh and NMDA, measured in an 8 s time window 120 s after the previous NMDA-delivery (ACh_NMDA). After acquiring pretreatment control (IP0) data, amnestic agents—scopolamine (Tocris) or MLA (Sigma-Aldrich)—were injected i.p., and test conditions 1–4 were repeated at three post-injection time points: at 10, 20 and 30 min after systemic administration of one of the amnestic agents (time points referred to as IP10, IP20 and IP30, respectively). Furthermore, a control measurement in each test condition was made immediately after the injection of scopolamine or MLA to control for any non-pharmacological effects of drug-administration (e.g., movement artifacts) on neuronal firing.

Both scopolamine and MLA were dissolved in physiological saline (0.9% NaCl) to a concentration of 1 mg/ml and were administered in a dose of 1 mg/kg (bw).

### Statistical Analyses

Statistical analyses were made on the firing rate (Hz) data measured in the previously defined time windows for the four test conditions (i.e., Sp, NMDA, ACh, ACh_NMDA).

Linear mixed-effects models were used for statistical analysis, in which the correlation of repeated measurements was taken into account by including a random intercept in the model. Statistical analyses were run in RStudio software (RStudio Inc., Boston, MA, USA) using R-packages *lme4* and *lmerTest* (Bates et al., 2015; Kuznetsova et al., 2015; R Core Team, 2015). After analyzing the main effects and interactions, the *lsmeans* package was used for *post hoc* comparisons, and *p* values were corrected using Holm’s method (Holm, 1979; Lenth and Hervé, 2015). Result plots were created using the *ggplot2 R* package (Wickham, 2009).

The following factors were defined in the statistical models for the analysis of firing responses. CONDITION referred to the four different test conditions defined earlier (levels: Sp, NMDA, ACh, ACh_NMDA). In the pretreatment control state, GROUP tested baseline differences between treatment groups (levels: pre-Scop, pre-MLA) before the administration of scopolamine or MLA. DRUG represented the two experimental groups corresponding to systemic treatment with scopolamine and MLA (levels: Scop and MLA, respectively). TIME examined the effect of systemic drug administration (i.e., scopolamine or MLA treatment) over the time course (levels: IP0, IP10, IP20, IP30).

We also calculated the distribution of recordings in which there was an increase, decrease or no change (threshold: ±20% change to control) in the firing rate 30 min after the systemic administration of scopolamine or MLA.

Further statistical analyses were conducted on the effects of combined iontophoretic treatments to establish whether simultaneous delivery of NMDA and ACh had simple additive or superadditive effect on the observed neuronal firing frequency, and how the amnestic agents scopolamine and MLA influenced such additive/superadditive effect.

Similar to the superadditivity analysis conducted in our laboratory as part of a behavioral pharmacological study (Trunk et al., 2015), the following null hypothesis was used:

$$H_{0}\;:\;(NMDA-Sp)+(ACh-Sp)=ACh\_NMDA-Sp$$

where, if *H*_0_ is kept (*p* > 0.05), then the combined effect of NMDA and ACh is additive, whereas a significantly higher (*p* < 0.05) value of ACh_NMDA − Sp compared to (NMDA − Sp) + (ACh − Sp) would indicate the superadditive nature of the simultaneous iontophoresis of the two compounds. Since the derived variables used in the superadditive analysis were calculated after subtracting the spontaneous firing rate from the firing rate value measured in a given test condition, we refer to the values of (NMDA − Sp) + (ACh − Sp) and ACh_NMDA − Sp as “relative firing rate change” for distinction from actual (absolute) firing rate values. The comparison of the sum of the mono-treatment effects and the combined effect was referred to in the statistical model as ADDITIVITY [levels: (NMDA − Sp) + (ACh − Sp) and ACh_NMDA − Sp, respectively].

### Histology

Positioning of the electrodes was primarily done using the stereotaxic coordinates and by looking for the electrophysiological properties typical to neurons intrinsic to the pyramidal cell layer of the hippocampal CA1 region. The reliability of the targeting was also confirmed at the end of four randomly selected experimental sessions by marking the recording sites with Chicago Sky Blue 6B dye (Sigma-Aldrich) for further histological examination. The dye was ejected through one of the surrounding microiontophoretic pipettes using negative constant current (−3 μA) for 20 min under deep anesthesia. Following histological marking, the animals were sacrificed and their brains were removed and immediately placed in 4% paraformaldehyde (Sigma-Aldrich). The brains were then sliced with a vibratome, and brain sections (50 μm thickness) were stained with Neutral Red (Sigma-Aldrich). Finally, electrode locations were identified under light microscopy. A typical example of a recording site labeling is depicted in Figure 1C.

## Results

The hippocampal neuronal firing activities of 15 animals were analyzed. Subjects were divided into two experimental groups: The systemic effects of scopolamine were studied in seven animals (three males), while the effects of MLA were examined in eight animals (three males). No statistical differences between female and male rats were found for either the baseline firing rate of the neurons or for the responses to any of the applied compounds. Final sample sizes were different for some measurement points due to missing values. In the scopolamine treatment group, the sample size for the ACh_NMDA test condition at IP0 was *N* = 6. In the MLA treatment group, the sample size for the NMDA and ACh_NMDA test conditions at each time point was *N* = 7.

### Simultaneous Delivery of NMDA and ACh Superadditively Increased the Firing Rate of CA1 Pyramidal Cells

**Figure 2:**
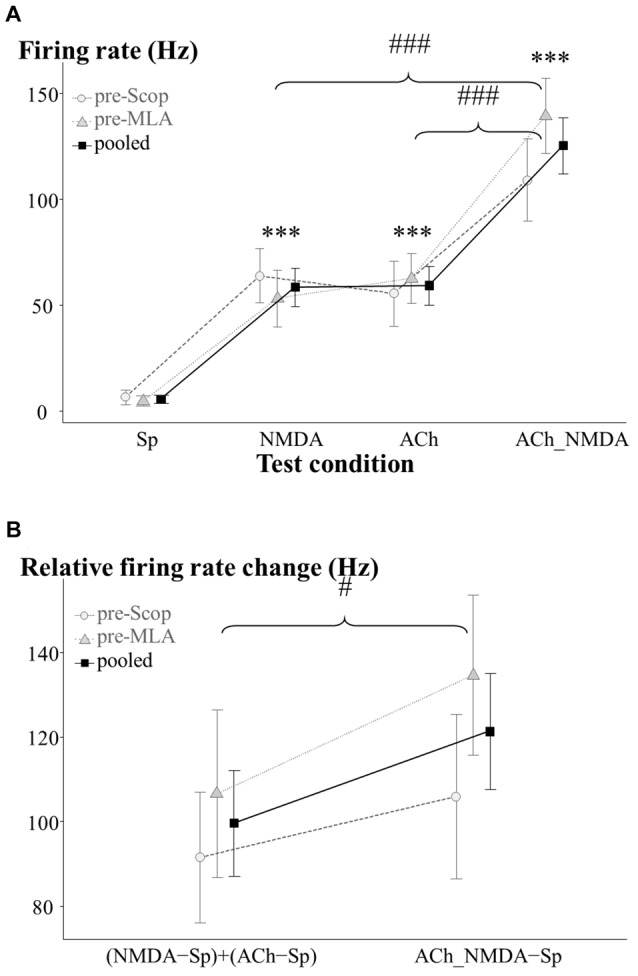
Firing rate responses of CA1 pyramidal cells to NMDA and ACh in the pretreatment control state (IP0). **(A)** Average firing rates under the four examined test conditions (*N* = 13–15). Sp: spontaneous firing rate, NMDA: NMDA-evoked firing rate, ACh: ACh-evoked firing rate, ACh_NMDA: firing rate evoked by simultaneous delivery of NMDA and ACh. **(B)** Testing the superadditive nature of the firing rate increase as a result of simultaneously delivered NMDA and ACh (*N* = 13). Note that in the superadditive analysis derived variables were used, which represent the firing rate change relative to the spontaneous firing activity. In both models, the two treatment groups (pre-Scop and pre-MLA) did not show any differences in their pharmacological responses to iontophoretically applied NMDA and ACh. Therefore, statistical comparisons were made using the pooled data, which was also plotted on the graphs. Significant differences vs. the spontaneous firing rate were marked with asterisks above the corresponding test condition: ***p < 0.001. Significant differences between other groups were marked with: ^#^*p* < 0.05, ^###^*p* < 0.001.

The results for hippocampal neuronal firing activity during the pretreatment control phase are summarized in Figure 2. The effects of iontophoretically applied NMDA and ACh were analyzed before systemic (i.p.) treatments with scopolamine and MLA.

As was expected, in the pretreatment control phase the two treatment groups (pre-Scop and pre-MLA) showed no differences in baseline firing rate or pharmacological response to iontophoretized ACh or NMDA (GROUP main effect: *F*_(1,12.5)_ = 0.17, *p* = 0.69, N.S.; GROUP × CONDITION: *F*_(3,36.2)_ = 0.97, *p* = 0.42, N.S.; see Figure 2A). Therefore, the control data for the two groups were pooled and a significant main effect of iontophoretic drug treatments was found (CONDITION: *F*_(3,36.2)_ = 39.51, *p* < 0.001). Microiontophoretic application of NMDA caused rapid firing rate increases, which typically lasted for 8 s. However, neurons showed slower responses to ACh (the effect was observable from 6.4 ± 1.3 s (mean ± SEM) after the start of delivery), and the firing rate increased gradually until reaching a relatively constant level of excitation. After the end of ACh-delivery, increased firing rate was still observable until 74.6 ± 5.7 s. Compared to the spontaneous activity, both NMDA and ACh significantly increased the firing rate (Sp: 5.6 ± 2.0 Hz, NMDA: 58.5 ± 8.9 Hz, ACh: 59.3 ± 9.2 Hz; Sp vs. NMDA: *p* < 0.001, Sp vs. ACh: *p* < 0.001), while the combination of NMDA and ACh led to a more pronounced increase in firing rate compared to the mono-treatments alone (ACh_NMDA: 125.4 ± 13.2 Hz; Sp vs. ACh_NMDA: *p* < 0.001, NMDA vs. ACh_NMDA: *p* < 0.001, ACh vs. ACh_NMDA: *p* < 0.001).

We further examined whether the combined effect of NMDA and ACh were superadditive compared to the independent (mono-treatment) effects of NMDA and ACh (Figure 2B). Again, the factorial analysis indicated no significant difference between the two experimental groups (GROUP main effect: *F*_(1,11)_ = 0.76, *p* = 0.40, N.S.; GROUP × ADDITIVITY: *F*_(1,11)_ = 0.61, *p* = 0.45, N.S.). Therefore, the data were analyzed independently of the subsequent systemic treatment (scopolamine or MLA). According to the analysis of the entire data pool, relative firing rate change in ACh_NMDA − Sp was found to be significantly higher than (NMDA − Sp) + (ACh − Sp) in the pretreatment control state (121.3 ± 13.6 Hz and 99.6 ± 12.5 Hz, respectively; ADDITIVITY main effect: *F*_(1,11)_ = 5.95, *p* < 0.05). Thus, the combined iontophoretic delivery of NMDA and ACh resulted in a significantly greater increase in firing rate compared to the summated effects of the mono-treatments. These results indicate a superadditive relationship in terms of the excitatory effects of simultaneously applied NMDA and ACh.

### Scopolamine Blocked the Tonic Excitatory Effect of ACh without Affecting the Synergism between ACh and NMDA

**Figure 3:**
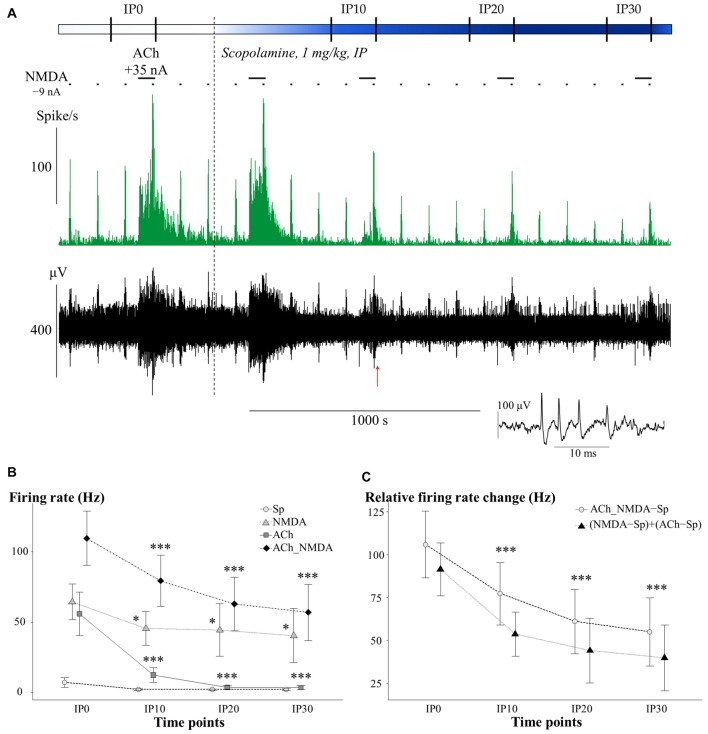
**(A)** Representative electrophysiological recording of CA1 hippocampal pyramidal cells before and after systemic scopolamine administration: firing rate histogram (top) and raw waveform data (bottom). Horizontal bars above the firing rate histogram indicate iontophoretic deliveries of NMDA and ACh. Inset shows one example of a typical complex spike. The red arrow under the trace indicate the position where the example spike was taken from. **(B)** Mean ± SEM plot of firing rate under different test conditions in the pretreatment control state (IP0) and at 10, 20 and 30 min after scopolamine administration (IP10, IP20, IP30, respectively; *N* = 6–7). Sp: spontaneous firing rate, NMDA: NMDA-evoked firing rate, ACh: ACh-evoked firing rate, ACh_NMDA: firing rate evoked by simultaneous delivery of NMDA and ACh. Since a significant interaction (CONDITION × TIME, see “Scopolamine Blocked the Tonic Excitatory Effect of ACh without Affecting the Synergism between ACh and NMDA” Section) was found, test conditions were analyzed separately and significant differences vs. the corresponding control (IP0) value in the given test condition were marked with: *p < 0.05; ***p < 0.001.** (C)** Changes in the sum of the mono-treatment effects [(NMDA − Sp) + (ACh − Sp)] and in the combined effect (ACh_NMDA − Sp) of iontophoretically applied NMDA and ACh over the course of time after scopolamine administration (*N* = 7). Since no interaction was found in ADDITIVITY × TIME, pooled data were used to assess significant changes (***p < 0.001) over the time course compared to IP0.

The effects of i.p. administered mAChR antagonist scopolamine on the firing rate of CA1 pyramidal neurons in different test conditions is presented in Figure 3. An additional representative electrophysiological recording is also shown in Supplementary Material (Supplementary Figure S1A). The four different test conditions were independently analyzed because the effect of scopolamine in distinct test conditions (Sp, NMDA, ACh and ACh_NMDA) was significantly different over the time course (CONDITION × TIME: *F*_(9,50.7)_ = 5.70; *p* < 0.001, see Figure 3B). While no significant difference was found in the spontaneous firing rate before and after i.p. injection of scopolamine (TIME: *F*_(3,18)_ = 2.19; *p* = 0.12, N.S.), the firing rate was decreased by at least 20% in six of the seven experimental sessions at 30 min after scopolamine administration (Table 1). On the other hand, scopolamine significantly decreased NMDA-evoked and ACh-evoked firing rate, as well as excitatory responses to the simultaneous iontophoretic delivery of NMDA and ACh, over the course of time (TIME main effects in the following conditions: NMDA: *F*_(3,18)_ = 3.73, *p* < 0.05; ACh: *F*_(3,18)_ = 11.21, *p* < 0.001; ACh_NMDA: *F*_(3,17)_ = 32.91, *p* < 0.001). At 30 min after scopolamine administration (IP30), the NMDA-evoked firing rate decreased from 64.6 ± 13.2 Hz to 39.9 ± 19.3 Hz (*p* < 0.05), and the ACh-evoked firing rate decreased from 51.8 ± 12.9 Hz to 3.0 ± 1.6 Hz (*p* < 0.001). The firing rate evoked by the simultaneous iontophoresis of NMDA and ACh (ACh_NMDA) decreased from 117.0 ± 18.1 Hz to 56.5 ± 20.1 Hz (*p* < 0.001). Scopolamine most profoundly inhibited the ACh-evoked responses, an effect that was observed in every individual experimental session, resulting in an average decrease in ACh-evoked firing rate to 5.8% of the pretreatment control values at 30 min after scopolamine injection (IP30). As a consequence, ACh alone no longer increased the firing rate compared to the spontaneous firing (CONDITION main effect at IP30: *F*_(3,18)_ = 5.78, *p* < 0.01; Sp − ACh: 1.5 ± 0.6 Hz vs. 3.0 ± 1.6 Hz; *p* = 0.93, N.S.). However, simultaneous delivery of NMDA and ACh still led to a significant increase in firing rate, even at 30 min after scopolamine administration, compared to the spontaneous firing rate as well as to the ACh-evoked firing response (IP30: Sp vs. ACh_NMDA: 1.5 ± 0.6 Hz vs. 56.5 ± 20.1 Hz, *p* < 0.05; ACh vs. ACh_NMDA: 3.0 ± 1.6 Hz vs. 56.5 ± 20.1 Hz, *p* < 0.05).

**Table 1:** Distribution of individual recording sessions, where firing rate under a given test condition increased (↑), decreased (↓), or did not change (Ø) by at least ±20% compared to pretreatment control at 30 min after scopolamine or methyllycaconitine (MLA) administration.

		Sp	NMDA	ACh	ACh_NMDA
Scopolamine	↑	1	0	0	0
	Ø	0	2	0	0
	↓	6	5	7	6
MLA	↑	4	3	3	0
	Ø	1	1	3	2
	↓	3	3	2	5

We furthermore analyzed whether the suppressed firing response to the simultaneous delivery of NMDA and ACh after scopolamine injection could be considered a simple additive decrease in the independent effects of NMDA and ACh, or whether it indicated the suspension of synergy between NMDA and ACh (Figure 3C). After scopolamine treatment, both (NMDA − Sp) + (ACh − Sp) and ACh_NMDA–Sp showed a similar decrease over the time course (TIME: *F*_(3,17)_ = 27.58, *p* < 0.001), and no interaction was found between ADDITIVITY and TIME (*F*_(3,17.1)_ = 1.27, *p* = 0.32, N.S.). Consequently, the combined effect of simultaneously delivered NMDA and ACh was significantly higher than the sum of the mono-treatment effects over the entire experiment (ADDITIVITY: *F*_(1,6.1)_ = 8.92; *p* < 0.05), even at 30 min after scopolamine administration [(NMDA − Sp) + (ACh − Sp) vs. ACh_NMDA − Sp: 40.0 ± 19.3 Hz vs. 55.0 ± 19.9 Hz, *F*_(1,6)_ = 5.78; *p* = 0.05]. The results indicate that the superadditive effect of the simultaneous delivery of NMDA and ACh were preserved even after scopolamine treatment.

Together, these results suggest that mAChRs play a substantial role in the ACh-mediated increase in tonic firing rate in the CA1 region. However, scopolamine did not influence the synergistic interaction between NMDA and ACh during simultaneous iontophoretic application of the two agonists.

### Methyllycaconitine Primarily Modified the Superadditive Effect of Simultaneously Applied NMDA and ACh

**Figure 4:**
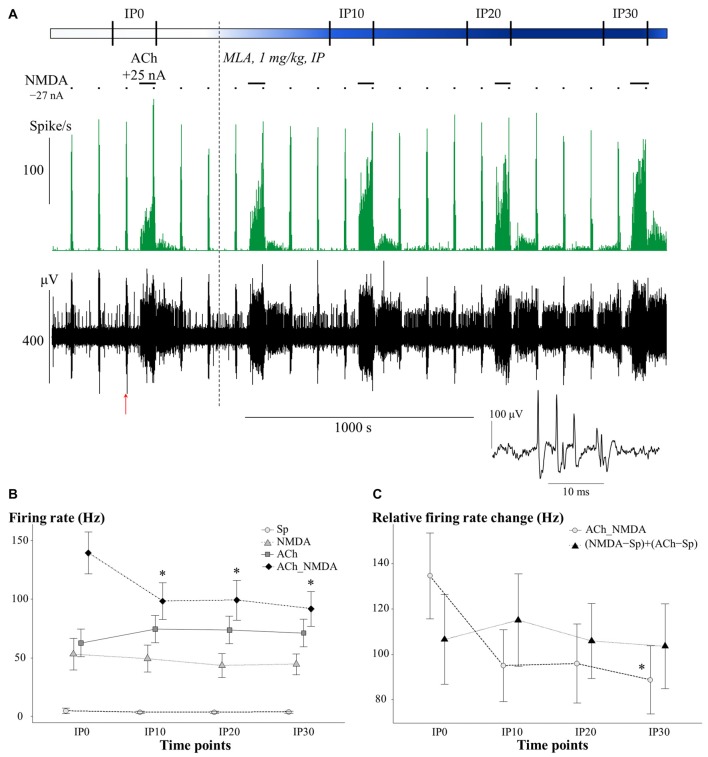
**(A)** Representative electrophysiological recording of CA1 hippocampal pyramidal cells before and after systemic MLA application: firing rate histogram (top) and raw waveform data (bottom). Horizontal bars above the firing rate histogram indicate iontophoretic deliveries of NMDA and ACh. Inset shows one example of a typical complex spike from the recording. The red arrow under the trace indicate the position where the example spike was taken from. **(B)** Mean ± SEM plot of firing rate under different test conditions in the pretreatment control state (IP0) and at 10, 20 and 30 min after the systemic administration of MLA (IP10, IP20, IP30, respectively; *N* = 7–8). Sp: spontaneous firing rate, NMDA: NMDA-evoked firing rate, ACh: ACh-evoked firing rate, ACh_NMDA: firing rate evoked by simultaneous delivery of NMDA and ACh. **(C)** Changes in the sum of mono-treatment effects [(NMDA − Sp) + (ACh − Sp)] and in the combined effects (ACh_NMDA − Sp) of iontophoretically applied NMDA and ACh over the course of time after systemic MLA administration (*N* = 7). Since in both models, significant interactions were found between main effects (CONDITION × TIME and ADDITIVITY × TIME, see “Methyllycaconitine Primarily Modified the Superadditive effect of Simultaneously Applied NMDA and ACh” Section), different test conditions and superadditivity variables were analyzed separately. Asterisks indicate significant differences vs. the corresponding control (IP0) values of the given test condition or superadditive variable (*p < 0.05).

The effects of systemically administered α7 nAChR antagonist MLA on the firing rate of CA1 pyramidal neurons under different test conditions is presented in Figure 4. An additional representative electrophysiological recording is also shown in Supplementary Material (Supplementary Figure S1B). Similar to the scopolamine treatments, the effects of MLA in the four test conditions were analyzed separately (Figure 4B), as a significant interaction was found between the different test conditions over the course of time (CONDITION × TIME: *F*_(9,59.1)_ = 3.02, *p* < 0.01). Systemically administered MLA exerted no significant effect on the spontaneous firing activity of the neurons (TIME: *F*_(3,21)_ = 0.38, *p* = 0.77, N.S.). In contrast to scopolamine, MLA did not modify the firing responses to separately iontophoretized NMDA or ACh (TIME main effects: NMDA: *F*_(3,18)_ = 0.47, *p* = 0.71, N.S.; ACh: *F*_(3,21)_ = 1.13, *p* = 0.36, N.S.). However, additional analysis of the distribution of individual recordings showed that systemic administration of MLA caused an approximately equal occurence of firing rate increases and decreases (Table 1). Furthermore, MLA significantly attenuated the firing rate increase evoked by simultaneous iontophoresis of NMDA and ACh (TIME main effect in ACh_NMDA condition: *F*_(3,18)_ = 3.55, *p* < 0.05). Thirty minutes after MLA application (IP30), the firing response of pyramidal cells to the combined treatment (ACh_NMDA) decreased to 65.8% of the pretreatment control (IP0 vs. IP30: 139.4 ± 17.7 Hz vs. 91.7 ± 14.7 Hz, *p* < 0.05; evoked firing activity decreased in almost every individual recording, see Table 1). Investigating the effects of iontophoretic drug application 30 min after MLA administration, we found that both NMDA and ACh, as well as their combined delivery, caused significant increases in firing rate compared to the spontaneous firing rate (CONDITION main effect at IP30: *F*_(3,19.2)_ = 23.12, *p* < 0.001; Sp: 3.6 ± 0.9 Hz, NMDA: 44.5 ± 8.9 Hz, ACh: 71.2 ± 11.7 Hz; ACh_NMDA: 91.7 ± 14.7 Hz; Sp. vs. NMDA: *p* < 0.01; Sp vs. ACh: *p* < 0.001; Sp vs. ACh_NMDA: *p* < 0.01). The firing rate evoked by the combination of NMDA and ACh was significantly higher than the NMDA-evoked firing activity at 30 min after MLA administration (NMDA vs. ACh_NMDA: 44.5 ± 8.9 vs. 91.7 ± 14.7 Hz, *p* < 0.01), but was not higher than the mono-treatment effect of ACh (ACh vs. ACh_NMDA: 71.2 ± 11.7 Hz vs. 91.7 ± 14.7 Hz, *p* = 0.09).

In contrast to the effects of scopolamine, MLA treatment caused a marginally significant interaction between ADDITIVITY and TIME (*F*_(3,18)_ = 2.97; *p* = 0.06; Figure 4C). MLA also attenuated the relative firing rate increase induced by simultaneous delivery of NMDA and ACh (TIME main effect on ACh_NMDA − Sp: *F*_(3,18)_ = 3.03, *p* = 0.06; IP0 vs. IP30: 134.6 ± 18.9 Hz vs. 88.8 ± 15.0 Hz, *p* < 0.05), while not affecting the sum of the mono-treatment effects (TIME main effect on (NMDA − Sp) + (ACh − Sp): *F*_(3,18)_ = 0.34; *p* = 0.80, N.S.). As a consequence, firing rate responses to the simultaneous delivery of NMDA and ACh were already similar to the sum of mono-treatments by 10 min after systemic MLA treatment (IP10); at this point, the combined effects were no longer superadditive. Analysis of the last post-injection measurement point (IP30) confirmed that the relative effect of simultaneously iontophoretized NMDA and ACh (ACh_NMDA − Sp: 88.8 ± 15.0 Hz) was not higher than the sum of the mono-treatment effects [(NMDA − Sp) + (ACh − Sp): 103.6 ± 18.7 Hz; *F*_(1,6)_ = 1.45; *p* = 0.27, N.S.)].

Together, these results indicate that antagonism of α7 nAChRs via systemic MLA treatment did not modify the tonic firing rate increase evoked by the local release of NMDA or ACh. However, MLA markedly decreased the neuronal responses to simultaneous delivery of NMDA and ACh, blocking the superadditive increase in firing rate that was observed in the pretreatment control state.

## Discussion

In the present experiments, *in vivo* extracellular spiking activity was recorded in the CA1 region of the hippocampus of anesthetized rats in combination with local microiontophoretic administration of NMDA and ACh. Subsequent systemic treatment with subtype-specific cholinergic antagonists (scopolamine or MLA) was used to assess the contribution of mAChRs or α7 nAChRs to the observed effects. The applied doses of the two antagonists were chosen as typical intermediate doses within the physiologically and behaviorally relevant dose range for rodents, according to previous studies from our laboratory and elsewhere (Matsumoto et al., 2001; Pichat et al., 2007; Huang et al., 2011; Andriambeloson et al., 2014; Bali et al., 2015).

### Predominant Role of mAChRs in the Modulatory Effects of ACh on the Tonic Activity of CA1 Pyramidal Neurons

During the pretreatment control phase, NMDA markedly increased the firing rate of CA1 pyramidal neurons, similar to earlier findings on locally delivered glutamatergic agonists (Biscoe and Straughan, 1966). Although chloral hydrate anesthesia reportedly attenuates glutamatergic neurotransmission (LacKamp et al., 2009), in our experiments, exogenously applied NMDA was clearly effective on CA1 neurons suggesting no substantial effects of the applied anesthetic on the evoked firing responses.

Our results confirm the generally accepted firing rate increasing effect of ACh on hippocampal neurons. However, net inhibitory actions of ACh were also recorded in hippocampal pyramidal cells in earlier *in vitro* experiments; these effects were mainly attributed to the indirect action of ACh through interneurons (Buhler and Dunwiddie, 2002). In contrast to the unspecific drug delivery by superfusion systems causing indirect inhibitory effects, in our experiments, the locally delivered ACh to the pyramidal cell layer of the CA1 region may not have been activated indirect inhibitory pathways. Therefore, in the discussion we will focus on the currently observed excitatory effects of ACh.

After the administration of mAChR antagonist scopolamine, a gradual and significant decline was observed in the firing responses to both NMDA and ACh over the course of time, while spontaneous baseline activity of the neurons was maintained at the control level. Previous studies similarly reported the lack of effects of mAChR blockage on the spontaneous firing activity of CA1 pyramidal neurons (Stewart et al., 1992), which can be explained by the already decreased neuronal activity and the concomitantly low ACh-levels caused by the general anesthesia (Marrosu et al., 1995; Gais and Born, 2004; Pagliardini et al., 2013). On the other hand, in our study, exogenously applied ACh failed to increase firing rate at 30 min after scopolamine administration, which indicates that the ability of iontophoretically delivered ACh to increase tonic firing rate was completely abolished by mAChR blockage. Earlier studies have also shown that the excitatory neuronal effects of ACh are blocked by mAChR antagonists (Biscoe and Straughan, 1966; Bland et al., 1974; Bird and Aghajanian, 1976; Cole and Nicoll, 1984) or by the knock-out of mAChRs (Dasari and Gulledge, 2011). A general inhibition of tonic neuronal activity originally maintained by appropriate extracellular ACh levels may also contribute to the memory encoding deficits observed after scopolamine administration.

Although a potent amnestic effect of α7 nAChR antagonist MLA has also been reported in behavioral studies (Vago and Kesner, 2007; Tinsley et al., 2011; Andriambeloson et al., 2014), no decrease in ACh-evoked firing rate was found in the present experiments after systemic injection of MLA. In line with our present observations, previous *in vitro* electrophysiological studies found that the ACh-evoked responses of pyramidal cells in the CA1 region were not sensitive to the blockage of nAChRs (Cole and Nicoll, 1984). Additionally, in the *in vitro* experiments of McQuiston and Madison (1999), most of the pyramidal neurons were not sensitive to direct nAChR stimulation, although a minority of the neurons showed fast (α7-type) nicotinic currents. Furthermore, GABAergic interneurons that are located and terminate in the pyramidal cell layer of the CA1 region also showed relatively lower sensitivity to nicotinic stimulation compared to interneurons in other CA1 layers. As we assume that iontophoretic delivery of ACh in our present experiments stimulated neurons predominantly in the stratum pyramidale, our findings regarding the ineffectiveness of MLA supports the general view that nAChRs do not play a critical role in the determination of tonic firing activity of CA1 pyramidal cells.

### Superadditive Effect of Simultaneously Delivered NMDA and ACh. Influence of Muscarinic and Nicotinic Blockage

In the pretreatment control phase, the simultaneous iontophoretic delivery of NMDA and ACh resulted in a significantly higher firing rate than the delivery of NMDA or ACh alone, due to superadditive increase of firing rate over the sum of mono-treatment effects. As the superadditive effect required the simultaneous activation of AChRs and NMDARs, we identified it as a manifestation of cholinergic potentiation of glutamatergic signaling. Similar enhancement of glutamatergic responses by ACh was previously shown with ACh and NMDA exogenously applied to hippocampal slices (Markram and Segal, 1990a), and with simultaneous stimulation of the medial septum and glutamatergic afferents to the CA1 region (Krnjević and Ropert, 1982; Markram and Segal, 1990b).

After the systemic administration of scopolamine, we observed a significant decrease in the firing rate response to the simultaneous delivery of NMDA and ACh. However, the decrease of the combined effect could be explained by the aforementioned decrease in the mono-treatment effects of ACh and NMDA; this was also confirmed by the superadditivity analysis. As such, simultaneous delivery of NMDA and ACh after scopolamine administration still resulted in a significantly higher firing response compared to the sum of the two mono-treatment effects. Moreover, the superadditive effect of the combined iontophoretic delivery of NMDA and ACh remained stable even at 30 min after systemic scopolamine treatment (IP30). Based on these results, we conclude that mAChR-mediated mechanisms that maintain tonic activity of pyramidal neurons, are not critically involved in the cholinergic potentiation of NMDA-evoked firing responses.

Previous *in vitro* studies have reported the facilitation of glutamatergic synapses through activation of mAChRs (Krnjević and Ropert, 1982; Markram and Segal, 1990a, b). However, there is also evidence for the opposing muscarinic action, namely a mAChR-mediated suppression of excitatory postsynaptic potentials that reduces the probability of transmitter release and the overall effectiveness of CA1 pyramidal neuron stimulation via glutamatergic afferents (Fernández de Sevilla et al., 2002). It is possible that, under *in vivo* conditions and after systemic injection of the non-specific mAChR antagonist scopolamine, these previously identified selective facilitatory and suppressive effects of mAChR activation may cancel each other out, resulting in the ineffectiveness of scopolamine on the superadditive interaction between NMDA and ACh.

Although the firing responses to the delivery of NMDA or ACh alone were not affected by systemically applied α7 nAChR antagonist MLA, the excitatory effect of their simultaneous iontophoresis was markedly attenuated by MLA, and the combined effect of NMDA and ACh was no longer higher than the sum of mono-treatment effects. Thus, MLA blocked the superadditive interaction between NMDA and ACh, suggesting that, in the present experimental arrangement, the substantial role in the cholinergic potentiation of glutamatergic neurotransmission was played by the α7 nAChRs and not by the mAChRs.

Results from earlier *in vitro* studies on hippocampal slices also suggest the involvement of α7 nAChRs in the physiological functioning of glutamatergic terminals, as it was reported that the frequency of excitatory postsynaptic currents was reduced by MLA (Banerjee et al., 2013), and that the α7 nAChR agonist S 24795 enhanced long-term potentiation in Schaffer-collaterals (Lagostena et al., 2008). Moreover, iontophoretically applied MLA inhibited NMDA-evoked excitation in the prefrontal cortex of monkeys during a working memory task (Yang et al., 2013), confirming the interaction between α7 nAChRs and glutamatergic synapses in cognitive performance.

### Possible Mechanisms for the Interaction of α7 nAChRs and NMDARs

The experimental methods used in the present study did not allow localization of the α7 nAChRs responsible for the observed ACh-mediated effects. However, one possible explanation for the mechanism of action could be the activation of presynaptic α7 nAChRs, which facilitate the release of excitatory amino acids from glutamatergic terminals in the frontal cortex and also in the hippocampus (Rousseau et al., 2005; Zappettini et al., 2010; Banerjee et al., 2012; Huang et al., 2014). Moreover, α7 nAChRs and NMDARs are co-localized on glutamatergic terminals, and their interaction appears also in the regulation of the presynaptic side, as it was demonstrated that α7 nAChRs agonist choline facilitated NMDA-evoked transmitter-release through the increased levels of intracellular [Ca^2+^] and the increased expression of presynaptic NMDARs (Zappettini et al., 2014). Furthermore, astrocytes may also play a role in the cholinergic enhancement of glutamatergic activation through different mechanisms. Patti et al. (2007) demonstrated the α7-nAChR-dependent release of glutamate from astrocytes, while Wang et al. (2013) showed that the activation of α7 nAChRs on glial cells resulted in the increased abundance of α-amino-3-hydroxy-5-methyl-4-isoxazolepropionic acid receptors (AMPAR) in the postsynaptic density of glutamatergic synapses. Although AMPARs were not directly targeted in our experiments, considering their permissive role in NMDAR functioning, the regulation of AMPARs is another possible mechanism for the effects of α7 nAChR activation on NMDA-induced responses. Moreover, *in vitro* studies suggested that α7 nAChRs may have an additional (or supplementary) permissive role on NMDAR activation similar to AMPARs (Levy and Aoki, 2002; Risso et al., 2004). Considering this and the fact that α7 nAChRs are also present on postsynaptic sites of glutamatergic synapses (Fabian-Fine et al., 2001; Yang et al., 2013), ACh acting on α7 nAChRs may provide a so called “permissive depolarization” of the postsynaptic membrane, thus increasing the number of responsive NMDARs.

In similar *in vivo* experiments carried out in the CA3 region, Huang et al. (2010) attributed the firing rate increasing effect of selective α7 nAChR agonists to presynaptic facilitation. The same mechanism might also be supposed in the CA1 region as a possible explanation for the present results. However, in that case, after ACh mono-treatment, the presynaptically increased glutamate levels should have resulted in the same firing rate increase as in the combined treatment (ACh_NMDA), which was not the case in our experiments. Therefore, presynaptic mechanisms do not fully explain the superadditive increase of NMDA-evoked firing rate when ACh is also present. In this regard, a postsynaptic mechanism (i.e., permissive depolarization) seems to be more probably, or at least partly involved in the observed effects.

### Conclusions and Further Implications

In summary, we conclude that mAChRs predominantly modulate the tonic neuronal activity of CA1 pyramidal cells, while the primary role of α7 nAChRs in cholinergic action is the potentiation of glutamatergic neurotransmission, which may be based on different presynaptic and/or postsynaptic mechanisms. Roles of ACh in maintaining appropriate tonic neuronal activity and in regulating glutamatergic signaling are of similar importance in memory, as both scopolamine and MLA are well known for their amnestic effects in cognitive behavioral tasks (Tinsley et al., 2011; Andriambeloson et al., 2014). Furthermore, the present results confirm the hypothesis that the cognitive enhancer potential of α7 nAChR activators may be related to glutamatergic transmission, and also explain our earlier behavioral results regarding the lower efficacy of α7 nAChR agonist PHA-543613 in case of the blockage of NMDARs (Bali et al., 2015). Further experiments are planned to clarify how known cognitive enhancers (e.g., nAChR agonists and allosteric modulators) may modify the tonic cholinergic excitation of neurons and potentiate NMDA-evoked responses.

## Author Contributions

ZKB and IH designed the experiment. ZKB and LVN performed electrophysiological recordings and post-mortem histological analysis. ZKB performed statistical data analyses. ZKB and IH wrote the manuscript. All authors approved the final manuscript.

## Conflict of Interest Statement

The authors declare that the research was conducted in the absence of any commercial or financial relationships that could be construed as a potential conflict of interest.

## Abbreviations

ACh: acetylcholine
AMPAR: α-amino-3-hydroxy-5-methyl-4-isoxazolepropionic acid receptor
GABA: gamma-aminobutyric acid
mAChR: muscarinic acetylcholine receptor
MLA: methyllycaconitine
nAChR: nicotinic acetylcholine receptor
NMDA: N-methyl-D-aspartic acid
NMDAR: N-methyl-D-aspartic acid receptor.
