# Supplementary material for: Alpha7 Nicotinic Acetylcholine Receptors Play a Predominant Role in the Cholinergic Potentiation of N-Methyl-D-Aspartate Evoked Firing Responses of Hippocampal CA1 Pyramidal Cells
Source: Front Cell Neurosci. 2017 Sep 5;11:271. doi: 10.3389/fncel.2017.00271 (PMC5591832; doi:10.3389/fncel.2017.00271)
Supplement: Supplementary file 1 [file Data_Sheet_1.docx]

Supplementary Material

Alpha7 nicotinic acetylcholine receptors play a predominant role in the cholinergic potentiation of N-methyl-D-aspartate evoked firing responses of hippocampal CA1 pyramidal cells

Zsolt Kristóf Bali, Lili Veronika Nagy, and István Hernádi*

*** Correspondence:** Corresponding Author: hernadi@ttk.pte.hu

## Supplementary Figures

##
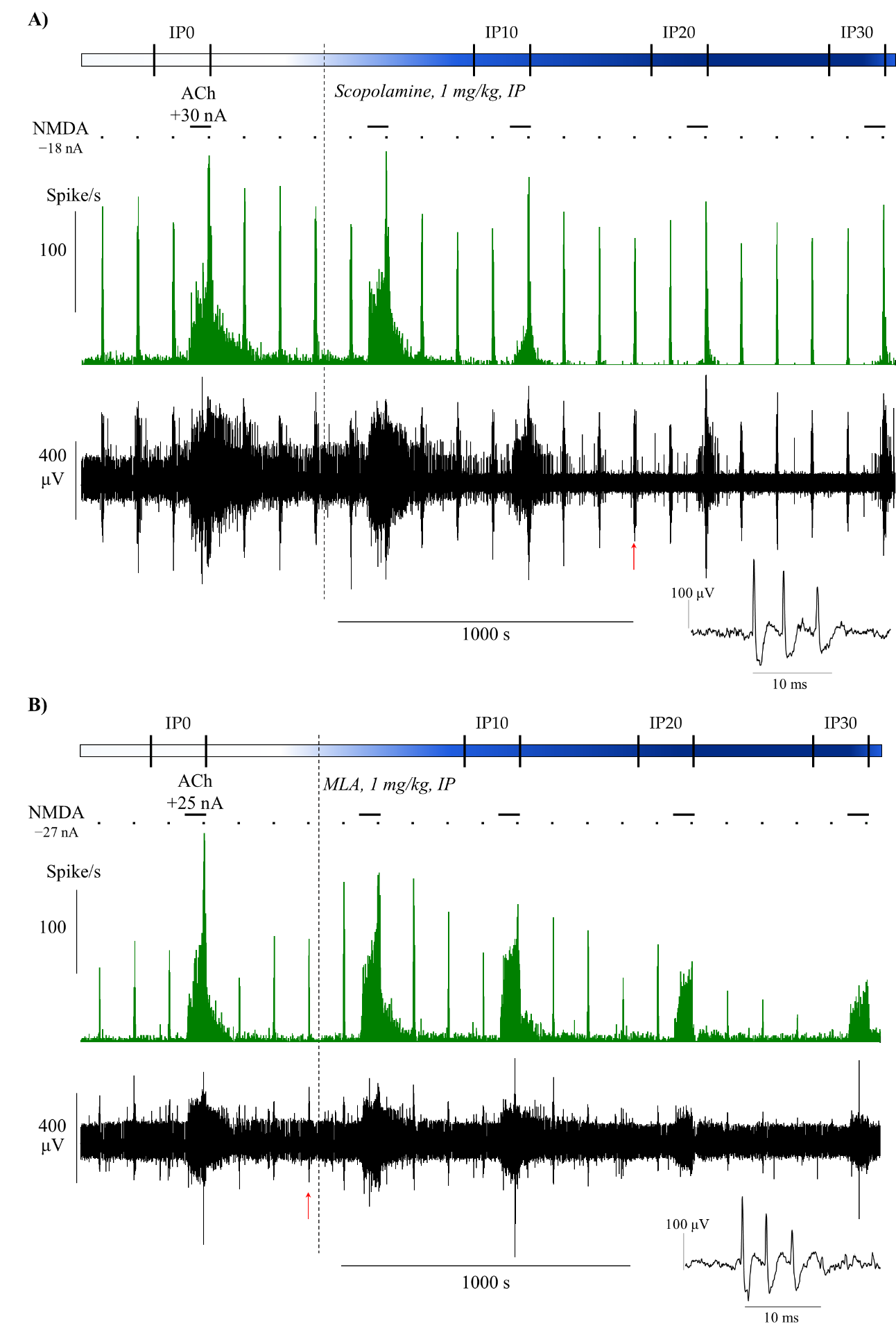


**Supplementary Figure 1.** **A)** Additional representative electrophysiological recordings of CA1 hippocampal pyramidal cells before and after systemic scopolamine **(A)** or MLA **(B)** administration: firing rate histograms (top) and raw waveform data (bottom). Horizontal bars above the firing rate histograms indicate iontophoretic deliveries of NMDA and ACh. Insets show one example of a typical complex spike. The red arrows under the corresponding trace indicate the position where the example spike waveform was taken from.
